# Supplementary figures and images for: Neodymium-Facilitated Visualization of Extreme Phosphate Accumulation in Fibroblast Filopodia: Implications for Intercellular and Cell–Matrix Interactions
Source: Int J Mol Sci. 2024 Oct 15;25(20):11076. doi: 10.3390/ijms252011076 (PMC11508255; doi:10.3390/ijms252011076)

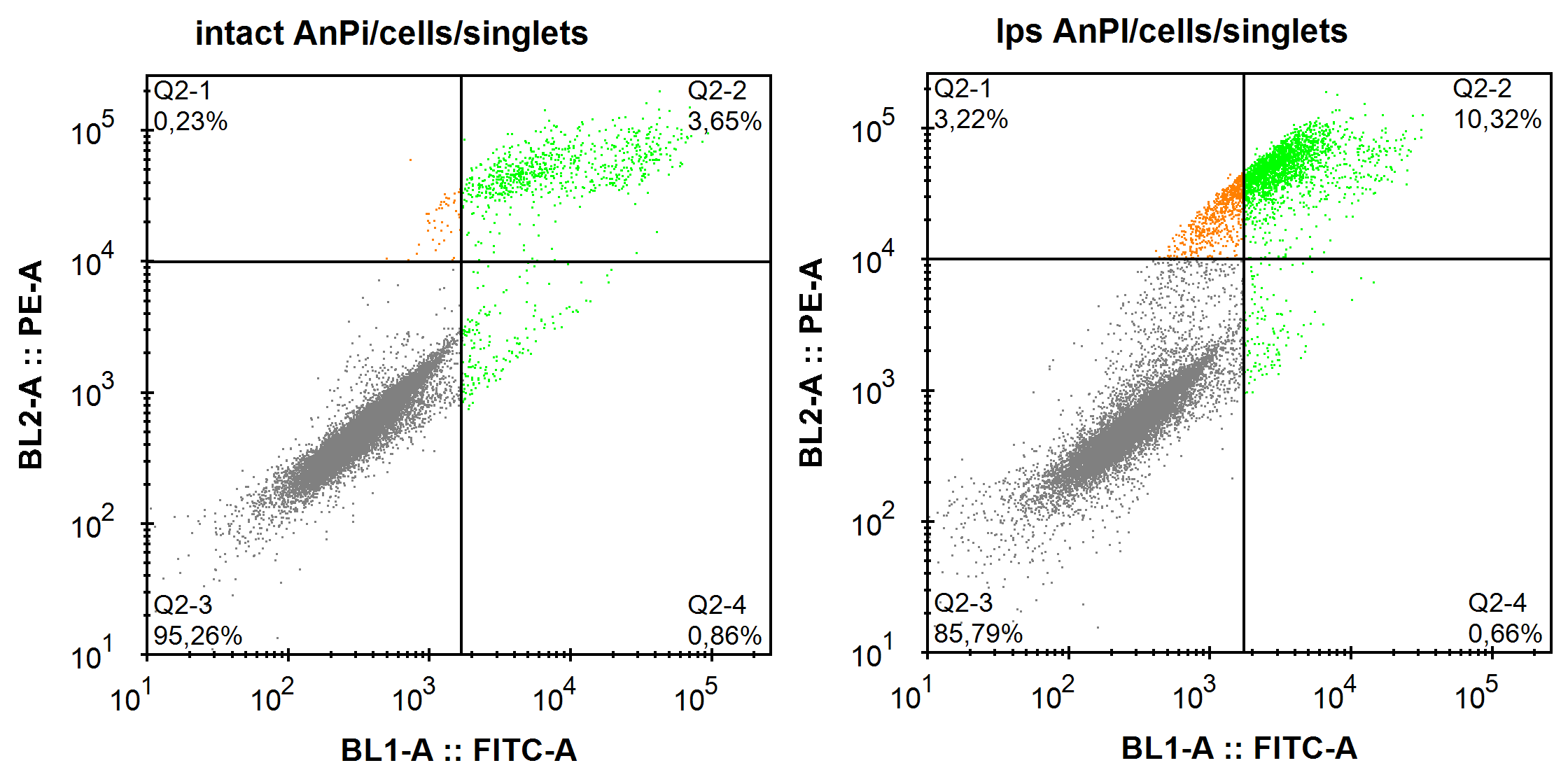

Supplement: Supplementary file 1 [file ijms-25-11076-s001.zip › FigS2.tif]
